# Esophagogastric Complications After Caustic Ingestion: A Case Report

**DOI:** 10.7759/cureus.26762

**Published:** 2022-07-11

**Authors:** Carla Valencia, Jose Prieto, Javier Jara, Priscila Pesantez

**Affiliations:** 1 Internal Medicine, Facultad de Medicina-Universidad del Azuay, Cuenca, ECU; 2 Internal Medicine, Loyola Medicine, MacNeal Hospital, Berwyn, USA; 3 General Surgery, Facultad de Medicina-Universidad del Azuay, Cuenca, ECU

**Keywords:** caustic injury, esophageal cancer, gastric outlet obstruction, esophageal stenosis, caustic ingestion

## Abstract

Ingestion of corrosive agents is a well-known factor in extensive GI tract injury. Either acids or alkalis can lead to significant immediate and long-term complications throughout the GI system. The ingestion of these substances in children is often accidental, however, it is typical that in adults is due to suicidal attempts.

A 25-year-old male with a history of suicidal attempt one month ago comes to the GI clinic due to unintentional weight loss of 19%, dysphagia of solids, emesis, and regurgitation. Evaluation with an upper gastrointestinal endoscopy was done which showed severe esophageal stenosis (90%), esophagitis Zargar 3a, and total pyloric stenosis. A dilation procedure was attempted, but complicated by severe bleeding; thus, the patient was hospitalized for a new attempt. During the second endoscopy, an esophageal dilation was successfully performed, but the pyloric dilation could not be completed. For that reason, a hepato-pancreato-biliary surgeon was consulted and a jejunostomy was performed for enteral nutrition.

Caustic agent ingestion can cause different types of injuries including laryngospasms, perforations, necrosis, and mediastinitis in the short term. On the other hand, esophageal stenosis (ES), gastric outlet obstruction (GOO), and esophageal cancer can appear in the long term. In this case, we highlighted the importance of prompt recognition, identification, and grading of the lesions to determine a better outcome and prognosis for the patient.

## Introduction

According to the American Association of Poison Control Centers (AAPCC), 200,000 people were exposed to household cleaning items since 2000 in the United States. This type of data can be difficult to measure in developing countries due to the underreporting of the patients and the lack of early emergency attention of the same [1].

Alkali or acid ingestion is a very common and studied cause of catastrophic gastrointestinal (GI) tract damage. These corrosive agents as commonly named can lead to significant immediate complications during their passage through the GI tract such as laryngospasms, perforations, necrosis, and mediastinitis, often leading to a fatal outcome in the first few hours of the incident. On the other hand, when the patients overcome this short-term complication, other pathologies such as esophageal strictures (ES), gastric outlet obstructions (GOO), squamous cell carcinoma, and adenocarcinoma of the esophagus may develop, generally after four to six weeks after the event [2].

The ingestion of these types of substances in children is often accidental. On the other hand, it is typical that the intake of corrosive agents in adults is due to suicidal attempts, especially in patients with psychiatric disorders and alcoholism [3]. For that reason, it is important to perform a holistic assessment of these patients, including a psychiatric evaluation, in order to improve the outcomes. In this article, we present a patient with multiple consequences of the autolytic event he had, and we highlight the importance of prompt recognition and effective treatment aiming to prevent them. The unique feature of this case is the rare co-occurrence of ES and GOO that represented a poor outcome for our patient with an extensive injury and a complicated course of treatment.

## Case presentation

**Figure 1:**
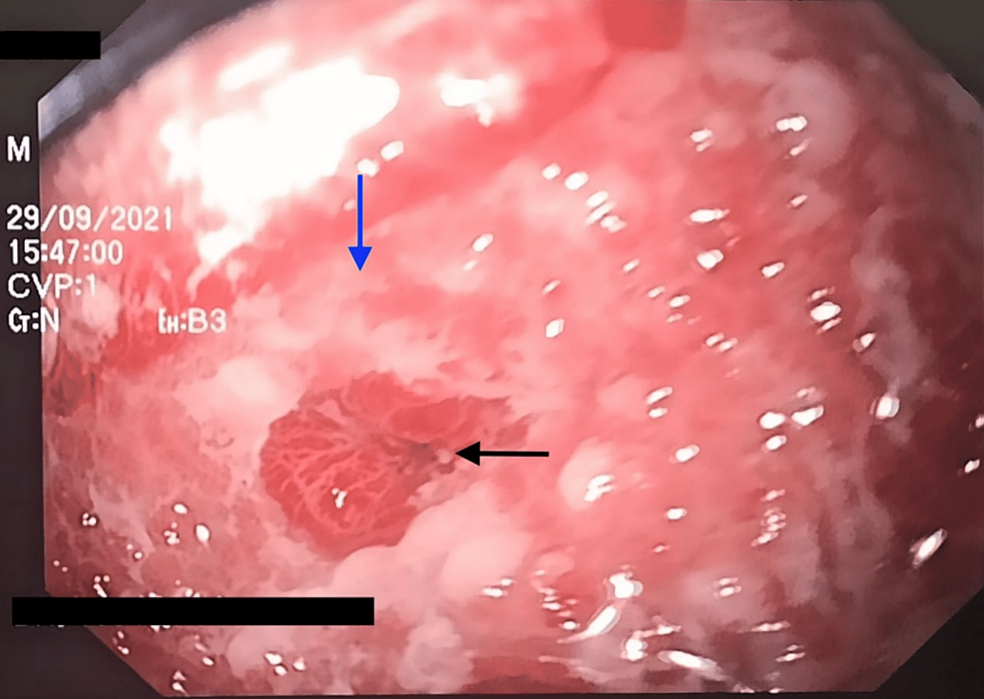
Esophageal stenosis with 90% of lumen obstruction (black arrow); esophagitis Zargar 3a (blue arrow)

**Figure 2:**
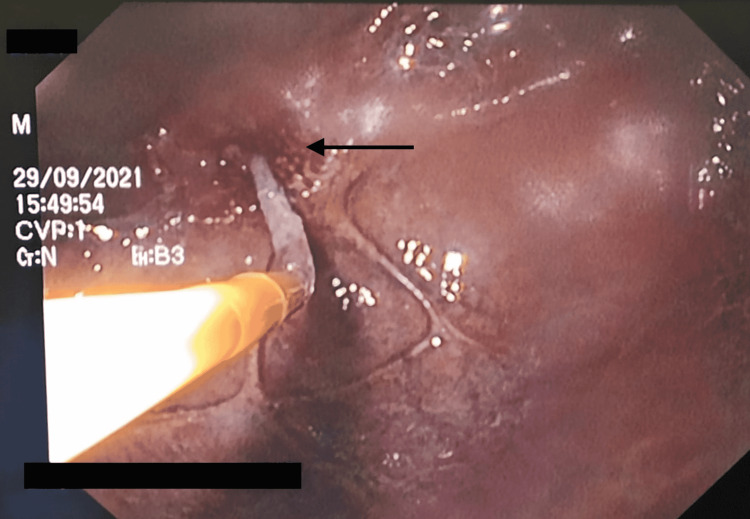
Total pyloric stenosis (arrow)

**Figure 3:**
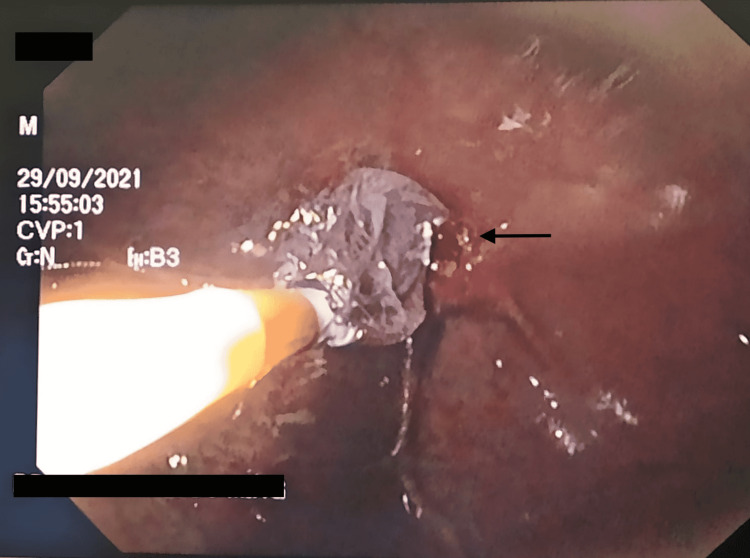
Failed attempt at pyloric dilation (arrow)

A 25-year-old male with a past medical history of alcoholism and negative history of psychiatric illness came to the gastroenterologist's office in the San Juan de Dios Hospital, Cuenca, Ecuador, due to dysphagia to solids, emesis, regurgitation, and unintentional weight loss of 19% for one month. The symptoms appeared after a suicide attempt after the ingestion of an unspecified quantity of Sodium hypochlorite. In the initial evaluation of the patient, his laboratories showed a normal complete blood count (CBC), slight hyponatremia of 134 mEq/ml with normal remaining metabolic panel. The attending physician decided to perform an endoscopic evaluation in which the following findings and procedures were reported: (1) esophagus - (i) severe esophageal stenosis with 90% of radius reduction at 22 cm of distance from the oral cavity (Figure 1, black arrow), (ii) esophagitis Zargar 3a caused by caustic ingestion (Figure 1, blue arrow), (iii) dilation of the stenotic esophagus with Savary dilators is attempted until 12 French, observing profuse bleeding. Treatment is started with 5 ml of adrenaline diluted with 1000 ml of saline controlling the bleeding. (2) Stomach - (i) severe mucosal congestion, (ii) total pyloric stenosis (Figure 2), and (iii) failed pyloric dilation with hydrostatic balloon (Figure 3). (3) Duodenum - not assessed due to total pyloric stenosis.

The gastroenterologist decided to hospitalize the patient, and a second upper gastrointestinal endoscopy was planned for pyloric dilation. The physical examination during admission reveals a hemodynamically stable adult with evident cachexia, bad general appearance, pallor, abdomen with augmented bowel sounds in all four quadrants, and tender to deep palpation in epigastrium.

During the planned second endoscopy, the following findings were reported: esophagus - altered caliber, size, and compliance. Not valuable gastroesophageal junction. The diaphragmatic impression is not valuable with total stenosis and ulcered mucosa that is dilated with Savary dilators and a hydrostatic balloon. Stomach - altered size and compliance, absent rugae occupied with retained gastric content. Ulcered, inflammatory cardias. Antrum with altered shape, size, and total stenosis. A dilation with a hydrostatic balloon is attempted repeatedly but is not successful and the procedure is suspended. 

After a failed attempt of pyloric dilation with a hydrostatic balloon, a hepato-pancreato-biliary surgeon is consulted. A jejunostomy for enteral nutrition was planned and performed without complications. Nutrition service was consulted, starting with 50% of caloric requirements, and was increased according to the patient’s tolerance. After the procedures, the patient was discharged from the hospital and is currently in follow-up.

## Discussion

As previously mentioned, there are several different outcomes that could happen after corrosive agent ingestion. As could be seen in the case presentation, our patient presented to the health care unit after one month of his suicidal attempt, thus, long-term complications of his alkali ingestion were expected. 

There are two types of strictures as a late outcome, the esophageal ones representing around 70-100% of patients, that appear usually two to three weeks after the event, and the gastric ones in 30-60% of individuals with Zargar scale 2b/3 injuries, presented after six to eight weeks (Table 1) [1,4]. These two complications occur simultaneously in about 20% of individuals as seen in the case presented. However, because of the rapid recognition of symptoms, this cooccurrence of complications is becoming rare and a sign of poor outcome [2]. Another severe complication is esophageal cancer (adenocarcinoma and squamous cell carcinoma), which is presented in 1-4% of patients after corrosive ingestion. This can appear after 15-30 years of the incident, with poor prognosis in patients with severe dysphagia and repeated balloon dilations [5].

**Table 1:** Endoscopic grading of caustic injuries - Zargar classification The table is adapted from De Lusong et al. (2017) [1] and Contini and Scarpignato (2013) [6].

Grade	Characteristics
0	Normal mucosa
1	Superficial edema and erythema
2a	Friability, hemorrhages, superficial ulcerations, exudates
2b	Deep discrete or circumferential ulcerations
3a	Grade 2a plus deep and circumferential ulcerations
3b	Extensive necrosis
4	Perforations

In order to determine the prognosis of the patient and the correct management, it is imperative to be aware of certain information about the corrosive agent ingested. First, inquiring about the physical form, either solid, liquid, or gas, will provide us with an insight into the potential extension of the injury. Second, the amount of the ingestion will determine the severity and could differentiate if the ingestion is accidental or voluntary [7]. The importance of determining the nature of the substance is because acids and alkalis have different pathophysiology and different extension of the injuries. Alkalis generally cause more extensive damage due to the liquefactive necrosis produced by the interaction of the substance, fats, and proteins of the body. Similarly, acids can cause damage but in less degree, a product of coagulative necrosis as a consequence of acid proteins production and clot formation. This coagulation process prevents further spreading of the lesions and less degree injuries. However, this is not always the case, and a thorough evaluation is recommended for all the patients, independently of the implicated agent [1].

After the correct identification of the features mentioned above, it is important to note that specific corrosive agents may cause other systemic effects such as hyponatremia, hypokalemia, and acidosis, especially with strong acids and alkalis, nevertheless, this is not always present. Moreover, it has been proven that the development of severe mucosal injury, renal failure, and mediastinitis have a worse prognosis [8]. For all the reasons explained above, the correct identification and prompt classification of the lesions using the endoscopic Zargar scale has great importance in these cases (Table 1).

Esophageal stenosis

Esophageal strictures are one of the most common late complications of caustic ingestion since the esophagus is the most proximal GI organ affected. Strictures or stenosis can occur in up to 70% of patients with Zargar 2b, and more than 90% in Zargar 3 lesions. Also, these lesions can appear often after the third week of ingestion, and even later by the eighth week [1]. 

As previously mentioned, the early identification of the incident and management of the lesions are key to properly treat patients and decrease the probability of complications. However, when complications occur, some other tools can be used, such as the presence of mediastinitis or chronic renal failure that can predict the mortality of the patients. Hollenbach et al. described that the patients over 65 years old and the ones who ingested alkali were more likely to develop severe lesions and had higher mortality. However, these data were obtained with a short sample, and studies with a greater cohort are suggested [8]. 

When an esophageal complication occurs, the gold standard treatment is the dilation of the stenosed section and surgery as the next step for refractory cases. The dilations vary between 40% and 90% in success, it could be complicated by perforation in 0-32% [9]. The most commonly used tools are the Bougie (Savary-Gilliard) and the balloon dilators. When the dilations are successful, they improve the dysphagia, reduce dilation frequency, and significantly improve the patient’s clinical condition [1]. 

Esophageal dilation has a recurrence rate of stenosis of approximately 40%. When this occurs, the clinicians can try alternative procedures such as stents, with the highest rate of resolution with metallic stents over biodegradable and plastic. However, when these conservative procedures fail, the last resource is surgical correction with a partial or total esophagectomy with gastric pull-up or colonic interposition [1]. 

Gastric outlet obstruction 

GOO is the second most common complication after caustic ingestion, with substances like sodium hydroxide, potassium hydroxide, and hydrochloric acid being the most commonly implicated. The extension of the injury either in the antrum or pylorus will determine the treatment. When mild or moderate stenosis is found, balloon endoscopic dilation is recommended, with a median number of sessions between 2 and 13. However, when the obstruction is severe, surgical intervention can be considered a first-line treatment, with a range of different interventions such as antrectomy, pyloroplasty, or gastrojejunostomy, depending on the location of the lesion [10]. These interventions when timely performed can reach a 0-10.7% rate of complications [2].

Esophageal cancer

It is well known that esophageal cancer (EC), either adenocarcinoma or squamous cell carcinoma, has a variety of risk factors that can affect its development. Risk factors such as smoking, alcohol, dietary factors, gastroesophageal reflux disease, and underlying esophageal diseases have an important role, and due to the variety of them, it is difficult to determine what was the exact cause of it when a patient develops cancer [11]. 

Despite these risk factors, if a patient develops EC and has a history of caustic ingestion, the probability of the origin of their cancer being caustic ingestion is 1000-fold more than the general population, and the prevalence can be from 7% to 30%, being squamous cell carcinoma more common than the adenocarcinoma [12,13]. It is important to mention that this is the latest complication of caustic injury and can appear after decades of the incident, especially in fibrotic areas and strictures where dilations and procedures were performed, thus the patient presented in this case report is at significantly high risk of developing it, and constant vigilance is advised [13].

## Conclusions

Caustic ingestion in the general population is an important issue for public health since it is associated with accidental ingestion in children and purposeful ingestion in adults. Because most of the time this is an emergent situation, the problem is often underreported, and the appropriate research on this topic is directly affected.

As we could see, the complications are common and vary in appearance. Thus, the timed and correct identification of late lesions is of vital importance in order to prevent further complications, improve the quality of life of the patients, and decrease their mortality. Endoscopy is the first-line treatment for most lesions, but it has to be carefully done in order to prevent further injuries. Also, surgery can be considered an important treatment since it can solve the complications once endoscopy fails, and it gives the patients better control of their symptoms. Finally, it has been proven that caustic ingestion is an important risk factor for esophageal cancer, thus, we recommend a periodical screening to detect soon these lesions and give the patients the best possible care.

We presented this case aimed to remind the medical community of the importance of prompt and adequate management of caustic ingestion and to highlight that there is a probability to have a concurrence of two or more complications that compromise the well-being of the patients.
